# *CD209 *in inflammatory bowel disease: a case-control study in the Spanish population

**DOI:** 10.1186/1471-2350-8-75

**Published:** 2007-12-10

**Authors:** Concepción Núñez, Javier Oliver, Juan Luis Mendoza, María Gómez-García, Carlos Taxonera, Luis M Gómez, Miguel A López-Nevot, Emilio G de la Concha, Elena Urcelay, Alfonso Martínez, Javier Martín

**Affiliations:** 1 Servicio de Inmunología Clínica, Hospital Clínico San Carlos, Madrid, Spain; 2 Instituto de Parasitología y Biomedicina, CSIC, Granada, Spain; 3 Unidad de Enfermedad Inflamatoria Intestinal, Hospital Clínico San Carlos, Madrid, Spain; 4 Unidad de Enfermedad Inflamatoria Intestinal, Hospital Virgen de las Nieves, Granada, Spain; 5 Servicio de Inmunología, Hospital Virgen de las Nieves, Granada, Spain

## Abstract

**Background:**

The etiology of Ulcerative Colitis (UC) and Crohn's Disease (CD), considered together as Inflammatory Bowel Diseases (IBD), involves environmental and genetic factors. Although some genes are already known, the genetics underlying these diseases is complex and new candidates are continuously emerging. The *CD209 *gene is located in a region linked previously to IBD and a *CD209 *functional polymorphism (rs4804803) has been associated to other inflammatory conditions. Our aim was to study the potential involvement of this *CD209 *variant in IBD susceptibility.

**Methods:**

We performed a case-control study with 515 CD patients, 497 UC patients and 731 healthy controls, all of them white Spaniards. Samples were typed for the *CD209 *single nucleotide polymorphism (SNP) rs4804803 by TaqMan technology. Frequency comparisons were performed using χ^2 ^tests.

**Results:**

No association between *CD209 *and UC or CD was observed initially. However, stratification of UC patients by *HLA-DR3 *status, a strong protective allele, showed that carriage of the *CD209*_G allele could increase susceptibility in the subgroup of *HLA-DR3*-positive individuals (p = 0.03 OR = 1.77 95% CI 1.04–3.02, *vs. *controls).

**Conclusion:**

A functional variant in the *CD209 *gene, rs4804803, does not seem to be influencing Crohn's disease susceptibility. However, it could be involved in the etiology or pathology of Ulcerative Colitis in *HLA-DR3*-positive individuals but further studies are necessary.

## Background

Inflammatory bowel diseases comprise two distinct entities, Crohn's disease (CD) and Ulcerative Colitis (UC). Both forms are characterized by a chronic inflammation of the intestine, but several clinical and immunological profiles differ between them. IBD is a multifactorial disease: environmental factors seem to be involved in disease onset in genetically susceptible individuals. Common susceptibility genetic components exist for both diseases as evidenced apparently by the higher risk of developing UC in relatives of patients with CD or *vice versa*, but also specific genes seem to play an important role in the development or course of each disease. For CD, *CARD15 *mutations are the main susceptibility factors described in Caucasian populations [1]. On the other hand, *HLA *genes show stronger effects on UC in our population, specifically *HLA-DR3 *seems to have a protective role in the development of this disease [2]. However, a complex genetic contribution exists in both diseases and new etiological genes remain to be discovered.

DC-SIGN (dendritic cell-specific ICAM3-grabbing non-integrin), also named CD209, is a type II membrane protein member of the C-type lectin receptor superfamily. It is expressed by dendritic cells and is involved in pattern recognition and immunoregulation [3]. The gene coding for this protein, *CD209*, is located in the region 19p13, where a linkage peak to IBD resulted from a genomewide scan performed in Canadian families [4]. A promoter variant of this gene, rs4804803, affects its transcriptional activity *in vitro *and it has been associated to susceptibility or severity to some infections [5]. Recently, this polymorphism has been also associated to susceptibility to a subset of celiac disease patients, another inflammatory condition sharing with IBD alterations in mucosal immunoregulation [6].

The aim of this study was to evaluate the relevance of the rs4804803 polymorphism in susceptibility to IBD.

## Methods

### Samples

A total of 515 Crohn's disease patients and 497 ulcerative colitis patients were collected from two Hospitals in Spain (Hospital Clínico San Carlos, Madrid; Hospital Virgen de las Nieves, Granada). All the patients were of Spanish white origin. Diagnosis of UC and CD was based on standard clinical, radiological, endoscopic, and histological criteria. Demographic and clinical characteristics of IBD patients are shown in Table 1. A total of 731 healthy individuals were used as controls, all of them ethnically matched, they were blood donors and staff members. Written informed consent was obtained from all the participants in the study, which was approved by the Ethical Committee of Hospital Clínico San Carlos and the Ethical Committee of Hospital Virgen de las Nieves.

**Table 1:** Clinical characteristics of IBD patients

**Ulcerative colitis**	
Sex (% M)	55
Mean (SD) age at onset (y)	35.1 (12.9)
Mean (SD) disease duration (y)	10.1 (7.1)
Smoking habits (%)	
Never	47.1
Ex/current	52.9
Extraintestinal manifestations (%)	44.4
Disease location (%)	
Left sided (including proctitis)	56.5
Extensive colitis/Pancolitis	43.5
Surgery (%)	13.8
Immunosupression (%)	24.4

**Crohn's disease**	

Sex (% M)	49.1
Mean (SD) age at onset (y)	28.3 (12.5)
<40 (A1)	81.2
>40 (A2)	18.8
Mean (SD) disease duration (y)	9.4 (6.3)
Smoking habits (%)	
Never	51.5
Ex/current	48.5
Disease location	
Ileal (L1)	43.2
Colonic (L2)	17.2
Ileocolonic (L3)	35.0
Upper GI tract (L4)	4.5
Disease Behavior	
Inflammatory (B1)	40.9
Stricturing (B2)	19.3
Perforating (B3)	39.8

Values are calculated only from those patients with available data. Vienna classification has been used to classify CD patients.

### Genotyping

One single nucleotide polymorphism (SNP), rs4804803, was analyzed by TaqMan technology as previously described [6]. The two SNPs studied in the *CARD15 *gene were analyzed using C__11717468_20 (R702W, rs2066844) and C__11717466_20 (G908R, rs2066845) assays on Demand from Applied Biosystems (Foster City, CA). The 1007fs deletion (rs2066847) in the same gene was studied by an Assay by Design, also from Applied Biosystems. *HLA-DR *typing had been previously performed in the UC and CD patients, respectively, as well as in controls, as described before [7].

### Statistical Analysis

Allelic and genotypic frequencies were compared between groups by means of χ^2 ^tests. Statistical analyses were performed using the statistical package EpiInfo v5.00 (CDC, Atlanta, USA).

## Results

No significant differences were observed when genotypic or allelic frequencies of the *CD209 *polymorphism studied were compared between CD or UC patients and controls (Table 2). However, the stratification of UC patients by *HLA-DR3*, a genetic factor protective for UC (p = 0.00002; OR = 0.48), showed association of the *CD209 *SNP with UC only in *HLA-DR3*-positive patients (Table 3). When *HLA-DR3*-positive patients were compared with controls a similar association emerged (p = 0.03 OR = 1.77 95% CI 1.04–3.02). Stratification of CD patients by the presence of *CARD15 *mutations did not show any significant result (data not shown). Similarly, no association was found after location was considered (left-sided vs. extensive colitis for UC, and ileal vs. colonic for CD).

**Table 2:** Genotypic and allelic frequencies of *CD209 *rs4804803 in CD and UC patients and in controls

*CD209*	Controls		CD		UC		Controls *vs*. CD	Controls *vs*. UC
	n = 731	%	n = 515	%	n = 497	%		
AA	446	0.61	293	0.57	294	0.59	Overall, p = 0.27	Overall, p = 0.54
AG	251	0.34	191	0.37	184	0.37	Carriers G, p = 0.14 OR = 1.19 (0.94–1.50)	Carriers G, p = 0.51 OR = 1.08 (0.85–1.37)
GG	34	0.05	31	0.06	19	0.04		
								
A	1143	0.78	777	0.75	772	0.78	G *vs*. A, p = 0.11 OR = 1.17 (0.96–1.41)	G *vs*. A, p = 0.76 OR = 1.03 (0.84–1.26)
G	319	0.22	253	0.25	222	0.22		

**Table 3:** Genotypic and allelic frequencies in Ulcerative Colitis patients stratified by *HLA-DR3 *status

*CD209*	*DR3*-positive		*DR3*-negative		*DR3*-positive vs. *DR3*-negative
	n = 66	%	n = 431	%	
AA	31	0.47	261	0.61	Overall, p = 0.08
AG	33	0.50	153	0.35	Carriers G, p = 0.04 OR = 1.73 (1.00–3.01)
GG	2	0.03	17	0.04	
					
A	95	0.72	675	0.78	G *vs*. A, p = 0.10 OR = 1.41 (0.91–2.16)
G	37	0.28	187	0.22	

## Discussion

We have studied the influence of the functional polymorphism rs4804803 of *CD209 *in susceptibility to UC and CD in the Spanish population. This gene seems to be increasing UC susceptibility in a subgroup of patients, those carrying *HLA-DR3*. It is known that *HLA-DR3 *is a strong protective factor for the development of the disease (*HLA-DR3 *allele: 7% in UC patients *vs*. 13% in controls, p = 5.7*10^-7 ^OR = 0.48 95% CI 0.36–0.63). Thus, *HLA-DR3*-positive patients, *i.e.*, those patients with minimal HLA genetic contribution to susceptibility, might present some additional susceptibility factors when compared with *HLA-DR3*-negative patients. Albeit the association described here barely reaches statistical significance, it seems rather interesting because a most similar effect of this gene has been recently described in other intestinal inflammatory pathology, celiac disease [6]. The *CD209 *gene was found to be increasing celiac disease susceptibility only in *HLA-DQ2*-negative patients, *i.e.*, those lacking the main genetic susceptibility factor described to celiac disease. Allele rs4804803_G seems to be involved in susceptibility to both diseases. A similar hypothesis to the one proposed in relation to celiac disease could be working in this case; the lower transcriptional activity consequence of the rs4804803_G allele would imply minor surveillance capacity of dendritic cells and probably increased persistence of pathogens in the gut. In these conditions, a chronic inflammation could ensue. A role of pathogens in initiation or maintaining of the inflammatory process in IBD has been repeatedly proposed [8]; CD209 could be one of the receptors involved in the recognition of some of those pathogens. This role of *CD209 *in UC is compatible with the stronger impact of environmental factors described in UC compared to CD [9].

However, the weak statistical significance obtained makes necessary further analyses to confirm the influence of the *CD209 *rs4804803_G allele in susceptibility to *HLA-DR3 *positive UC patients in other populations and specially to investigate the involvement of this functional polymorphism in other chronic inflammatory conditions, mainly in those in whose origin pathogens could be involved. As a matter of fact, it is known that several common genes are underlying different autoimmune diseases [10].

Ulcerative colitis has been often proposed to be a heterogeneous group of diseases included together because of their clinical similarities [11]. Therefore, it is not surprising to find particular genes affecting only a previously well-defined genetic subset of patients. This kind of findings could be rewarding from a therapeutic perspective since patients potentially responding to a specific treatment could be previously selected.

## Conclusion

We reported a new susceptibility factor affecting to an ulcerative colitis subgroup of patients, those carrying *HLA-DR3*. Because CD209 is involved in pathogen recognition and the rs4804803_G *CD209 *allele results in lower transcriptional activity, the presence of this allele might generate an increased persistence of pathogens in the gut and consequently a chronic inflammation.

## Abbreviations

caspase recruitment domain 15 (CARD15); Crohn's Disease (CD); Human leukocyte antigen (HLA); Inflammatory Bowel Diseases (IBD); Single nucleotide polymorphism (SNP); Ulcerative Colitis (UC).

## Competing interests

The author(s) declare that they have no competing interests.

## Authors' contributions

CN and JA carried out the genotyping of the samples, participated in the statistical analysis and drafted the manuscript. LMG participated in the statistical analysis and revised the manuscript. JLM, MGG, CT and MALP made the diagnosis, collaborated in collection of samples and revised critically the manuscript. EGC coordinated the study and critically revised the manuscript. EU, AM and JM conceived the study, participated in the statistical analysis and completed the writing of the manuscript.

## Pre-publication history

The pre-publication history for this paper can be accessed here:


